# Platform trials: key features, when to use them and methodological challenges

**DOI:** 10.5694/mja2.52711

**Published:** 2025-06-22

**Authors:** Robert Mahar, Steve Webb, Ian Marschner, Andrew B Forbes, Katherine J Lee

**Affiliations:** ^1^ University of Melbourne Melbourne VIC; ^2^ Australian and New Zealand Intensive Care Research Centre Monash University Melbourne VIC; ^3^ National Health and Medical Research Council Clinical Trials Centre University of Sydney Sydney NSW; ^4^ Monash University Melbourne VIC; ^5^ Murdoch Children's Research Institute Melbourne VIC

**Keywords:** Biostatistics, Clinical trials as topic, Randomized controlled trial as topic

A revolution in evidence‐based medicine is currently underway that is being driven by significant innovation in clinical trial design.^1^ At the vanguard of this revolution is the platform trial.^2^, ^3^ A working definition of a platform trial is that of a randomised trial design that compares at least one intervention to a control and that has the capacity to add and remove interventions over time according to rules defined in a master (or core) protocol. In this way, platform trials can investigate multiple research questions under a shared and ongoing trial infrastructure, leading to operational efficiencies and improved allocation of resources. A recently published review identified, as of July 2022, 127 registered platform trials with a combined 823 arms, at either an ongoing (67.7%), completed (20.5%), discontinued (7.9%), planning (3.1%), or unclear (0.8%) stage of implementation, with most being started within the last five years.^4^ In Australia, multiple platform trials serve as examples of the possibilities of these designs, and a list of platform trials currently running in Australia can be found on the Australian Clinical Trials Alliance website (https://clinicaltrialsalliance.org.au/resource/adaptive‐platform‐trial‐operations‐special‐interest‐group‐trial‐summaries/).

Typically, platform trials include statistical adaptations, such as early stopping rules, that are used to make an early conclusion about treatment efficacy,^5^ or response adaptive randomisation.^6^ These features can reduce sample sizes, lead to preferential allocation to the best‐known treatments, and expedite conclusions. However, a platform trial may not include such adaptations. For example, platform trials may randomise all treatments in parallel batches or sequentially based on pre‐specified sample sizes. The “platform” in platform trials can be thought of as the infrastructure component (ie, a flexible protocol implemented under a shared infrastructure) that may have many possible statistical features (eg, multistage early stopping or factorial design).

To illustrate, we consider a platform trial that starts as a simple two‐arm trial with pre‐specified stopping rules and statistical design that allows the adding and dropping of treatments (Box). In this example, stopping rules are included for specific interventions, including an efficacy rule (ie, an intervention is better than control), and a futility rule (ie, the intervention is ineffective or is unlikely to be shown to be effective), with the set of control and interventions changing over time in response to these rules. Clearly, this trial has operational efficiencies compared with the alternative of conducting three independent two‐arm trials.

More complicated platform trial protocols might expand on the simple design shown in the Box, leading to additional efficiencies, for example, by imposing subgroup‐specific stopping rules (possibly using different interventions in different subgroups eg, adults, paediatrics). Some platform trials are multifactorial, simultaneously evaluating different classes of treatments available (“domains”, eg, antibiotics, steroids), with each participant randomised to a combination of treatments from each domain, contributing information to multiple research questions rather than to a single question, as would be the case in separate randomised trials. The Randomised, Embedded, Multifactorial Adaptive Platform for Community Acquired Pneumonia (REMAP‐CAP) is a well known example of a trial that includes response adaptive randomisation, statistical early stopping rules and, at the time of writing, 66 interventions (across 18 domains) are either currently active or completed, having so far achieved about 24 500 patient randomisations (over about 14 000 patients).^7^, ^8^

Box 1 **An example platform trial**

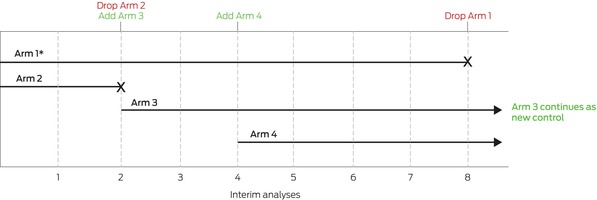

This platform trial starts as a two‐arm trial of Arm 1 and Arm 2 (* indicates the control arm). At the second interim analysis, Arm 2 is stopped (eg, for futility), and Arm 3 is added. At the fourth interim analysis, Arm 4 is added. At the eighth interim analysis, Arm 3 is shown to be efficacious relative to Arm 1 (ie, better than control), therefore Arm 1 is dropped and Arm 3 continues as the new control.

## When to use a platform trial

Platform trials are most useful when there is the potential to expand the trial to incorporate new interventions, new domains or new population subgroups, either as they become operationally or statistically feasible, or in response to changing clinical practices or emerging evidence.

To satisfy these criteria, platform trials are best suited when there is considerable clinical equipoise around best practice for a given medical condition that is likely to continue even in the presence of ongoing discoveries. For example, multiple platform trials emerged during the early stages of the coronavirus disease 2019 (COVID‐19) pandemic where there was considerable uncertainty around COVID‐19 treatments and a global need to expedite research.^4^, ^9^

Medical conditions with very few existing or emerging candidates for treatment, either because there are well established and effective treatments or no pipeline of candidates, are unlikely to be suitable for platform trials.

## Methodological challenges

Platform trials require specialised expertise for design, implementation and analysis. Beyond the usual considerations, such as sample size feasibility, operational resources, end points of interest, minimal clinically important differences, and burden of disease, investigators need clear scientific rationales to motivate the design, including the number and type of interventions to include, potential subgroups of interest and their heterogeneity, and the number, type and timing of interim analyses and associated early stopping rules. Clinicians and statisticians must work together to define and justify the master protocol and, if warranted,^10^ any adaptive strategies, including appropriate stopping rules,^5^ when and how to add or drop interventions,^11^ whether to use response adaptive randomisation,^6^ and managing sources of confounding arising from adaptations occurring when background risk or standard of care changes over time.^12^, ^13^

Depending on the complexity of the design, the trial management and documentation burden of platform trials, relative to traditional trials, is substantial.^14^ Platform trials are more dynamic than conventional trials, requiring additional resources devoted to ensuring timely data delivery for frequent scheduled analyses, operational challenges of implementing new interventions including ethics, governance and site training, ensuring complicated consent processes are robust and easy to explain, timely reporting of conclusions, and assessing eligibility where multiple domains are used. In addition to the core trial management group, trial steering committee, and data monitoring and safety committee, platform trial governance may require additional advisory, oversight and operational committees, including a statistical advisory committee to navigate the additional statistical complexities and a dedicated analytical team of statisticians. These groups need to continually interact throughout the life of the platform trial to ensure trial integrity. Robust communication firewalls are required to mitigate operational biases to prevent unblinding. Separate committees may also be required to inform and guide the design with respect to different interventions, domains or subgroups, often encapsulated in substantial appendices to the master protocol. The statistical design requires comprehensive pre‐specification, and the implementation of that design may require regular, blinded advice to the analytical team in the form of interim analysis guides, and intervention‐ or domain‐specific statistical analysis plans.

Where the statistical design is incongruent with well established frequentist adaptive designs, determining the statistical power of platform trial designs requires computer simulations, which involves running many thousands of hypothetical trials across many possible scenarios and evaluating how they perform on average. These simulations are required to ensure that the design has satisfactory type I error (false‐positive rate) and power. Simulations require time, expertise and substantial initial investment in the statistical team. Simulations are usually iterative, with multiple rounds of simulation, discussions with clinical investigators, and revision before arriving at a design that is statistically and clinically acceptable. This process can take many months at the design stage of the trial, but also needs to be updated during the trial when adaptations to the trial protocol are made.

Because of their complexity and their integration of multiple research questions, platform trials are best developed within strong research collaborations, existing trial networks, or with strategic consensus on their need within the clinical community. Importantly, they rely on having a critical mass of statistical expertise to design and implement the trial, particularly if there are frequent and ongoing interim analyses planned.

Statistical innovations may improve statistical inference and reduce sample sizes, such as the use of hierarchical statistical models that allow information to be “borrowed” across either interventions or population subgroups.^15^ Many platform trials use Bayesian statistical methods because of their flexibility and principled framework for information borrowing, sequential analysis, stopping rule formulation, and probabilistic interpretation of efficacy.^16^ Bayesian methods require specific expertise from the statistical team, extensive simulations for sample size planning, and familiarity with the methods among clinical investigators, which may require substantial training and education.

Despite the economies of scale that platform trials can achieve when adding new interventions or domains, they require a much greater initial investment compared with traditional designs. Funding bodies, both in Australia and internationally, are now making targeted calls for such upfront investment to develop platform trials. However, the considerably larger initial set‐up cost is only justified when shared across enough questions. Corresponding methodological research into platform trial designs is important to evaluate and improve ongoing and future platform trials.

## Conclusions

Globally, platform trials have emerged as a valuable, albeit operationally and statistically complex, design innovation that offers investigators a flexible, customisable and efficient way to rapidly contribute to the evidence base. Platform trials are suitable for many different diseases but require substantially more expertise and resources compared with traditional randomised trials and should only be considered when the scope and nature of the research questions are sufficient to justify the additional resources.

## Open access

Open access publishing facilitated by The University of Melbourne, as part of the Wiley – The University of Melbourne agreement via the Council of Australian University Librarians.

## Competing interests

Steve Webb is an adviser (but neither director nor shareholder) to Empiric Health, which is a for‐profit commercial entity that aims to utilise adaptive trials and adaptive platform trial methods to improve the efficiency and value of clinical trials. He has not received any income from Empiric Health but expects to do so.

## Provenance

Commissioned; externally peer reviewed.

## Author contribution statement

Mahar R: Conceptualization, writing – original draft, writing – review and editing. Webb S: Conceptualization, writing – review and editing. Marschner I: Conceptualization, writing – review and editing. Forbes A: Conceptualization, writing – review and editing. Lee K: Conceptualization, writing – review and editing.
